# Primary health care utilisation and delivery in remote Australian clinics during the COVID-19 pandemic

**DOI:** 10.1186/s12875-024-02485-3

**Published:** 2024-07-05

**Authors:** Supriya Mathew, Michelle S. Fitts, Zania Liddle, Lisa Bourke, Narelle Campbell, Lorna Murakami-Gold, Deborah J Russell, John S. Humphreys, Bronwyn Rossingh, Yuejen Zhao, Michael P. Jones, John Boffa, Mark Ramjan, Annie Tangey, Rosalie Schultz, Edward Mulholland, John Wakerman

**Affiliations:** 1 Menzies School of Health Research, Charles Darwin University, Alice Springs, Australia; 2 Institute for Culture and Society, Western Sydney University, Parramatta, NSW Australia; 3 Department of Rural Health, The University of Melbourne, Shepparton, VIC Australia; 4 Flinders Rural and Remote Health Northern Territory, College of Medicine and Public Health, Flinders University, Darwin, NT Australia; 5 Poche SA + NT, Flinders University, Alice Springs, Australia; 6 School of Rural Health, Monash University, Bendigo, VIC Australia; 7 Miwatj Health Aboriginal Corporation, Nhulunbuy, NT Australia; 8 Northern Territory Department of Health, Darwin, NT Australia; 9 School of Psychological Sciences, Macquarie University, North Ryde, NSW Australia; 10 Central Australian Aboriginal Congress, Alice Springs, Northern Territory, Australia; 11 Top End Population and Primary Health Care, Northern Territory Government, Casuarina, NT Australia; 12 Ngaanyatjarra Health Service, Alice Springs, Northern Territory, Australia; 13 Independent researcher, Minyerri, NT Australia

**Keywords:** Healthcare access, COVID, First Nations people, Aboriginal people, Clinic use

## Abstract

**Introduction:**

The COVID-19 pandemic period (2020 to 2022) challenged and overstretched the capacity of primary health care services to deliver health care globally. The sector faced a highly uncertain and dynamic period that encompassed anticipation of a new, unknown, lethal and highly transmissible infection, the introduction of various travel restrictions, health workforce shortages, new government funding announcements and various policies to restrict the spread of the COVID-19 virus, then vaccination and treatments. This qualitative study aims to document and explore how the pandemic affected primary health care utilisation and delivery in remote and regional Aboriginal and Torres Strait Islander communities.

**Methods:**

Semi-structured interviews were conducted with staff working in 11 Aboriginal Community-Controlled Health Services (ACCHSs) in outer regional, remote and very remote Australia. Interviews were transcribed, inductively coded and thematically analysed.

**Results:**

248 staff working in outer regional, remote and very remote primary health care clinics were interviewed between February 2020 and June 2021. Participants reported a decline in numbers of primary health care presentations in most communities during the initial COVID-19 lock down period. The reasons for the decline were attributed to community members apprehension to go to the clinics, change in work priorities of primary health care staff (e.g. more emphasis on preventing the virus entering the communities and stopping the spread) and limited outreach programs. Staff forecasted a future spike in acute presentations of various chronic diseases leading to increased medical retrieval requirements from remote communities to hospital. Information dissemination during the pre-vaccine roll-out stage was perceived to be well received by community members, while vaccine roll-out stage information was challenged by misinformation circulated through social media.

**Conclusions:**

The ability of ACCHSs to be able to adapt service delivery in response to the changing COVID-19 strategies and policies are highlighted in this study. The study signifies the need to adequately fund ACCHSs with staff, resources, space and appropriate information to enable them to connect with their communities and continue their work especially in an era where the additional challenges created by pandemics are likely to become more frequent. While the PHC seeking behaviour of community members during the COVID-19 period were aligned to the trends observed across the world, some of the reasons underlying the trends were unique to outer regional, remote and very remote populations. Policy makers will need to give due consideration to the potential effects of newly developed policies on ACCHSs operating in remote and regional contexts that already battle under resourcing issues and high numbers of chronically ill populations.

**Supplementary Information:**

The online version contains supplementary material available at 10.1186/s12875-024-02485-3.

## Introduction

Primary Health Care (PHC) plays a crucial role in delivering health care, especially as geographic remoteness increases and population density decreases where there is high need to optimise the use of limited specialist services. Around 10% of the Australian population and over 34% of Aboriginal and Torres Strait Islander people (hereafter respectfully referred to as Indigenous people) live in outer regional, remote or very remote locations [1]. Indigenous people living in remote and very remote Australia experience a higher burden of injury and disease, shorter lives, and poorer access and use of health services compared to urban residents [2]. This highlights the importance of strong PHC delivery and effective utilisation of PHC services offered to community members [3]. The prevention and management of chronic conditions is a substantial challenge faced by the Australian health system [4]. Chronic conditions, the leading cause of illness, disability and death in Australia, consume a huge proportion of the Australian health budget (more than a third of the national health budget spent on Primary Health Care (PHC) [5]. About 80% of the mortality gap between Aboriginal and non-Indigenous people aged 35 to 74 years is due to chronic diseases [6].

Australian populations living inmost remote and very remote communities are serviced by government run and/or Aboriginal Community-Controlled Health Services (ACCHSs), while some remote and outer regional populations additionally have access to private practices. In remote and very remote clinics, staffing usually comprises of Remote Area Nurses (RANs), Aboriginal Health Practitioners (AHPs), administrative staff (e.g. receptionists, drivers and cleaners), and resident and visiting medical and allied health professionals (e.g. General Practitioners (GPs), medical specialists, allied health professionals) [7]. Many very remote clinics operate with 2 nurses in a clinic [8]), experience high staff turnover [9], which affects continuity of care. Community members prefer long term staff with established, trusting relationships who are culturally and clinically competent [10], though seldom do staff stay long enough in remote communities to foster development of such relationships.

**Fig. 1:**
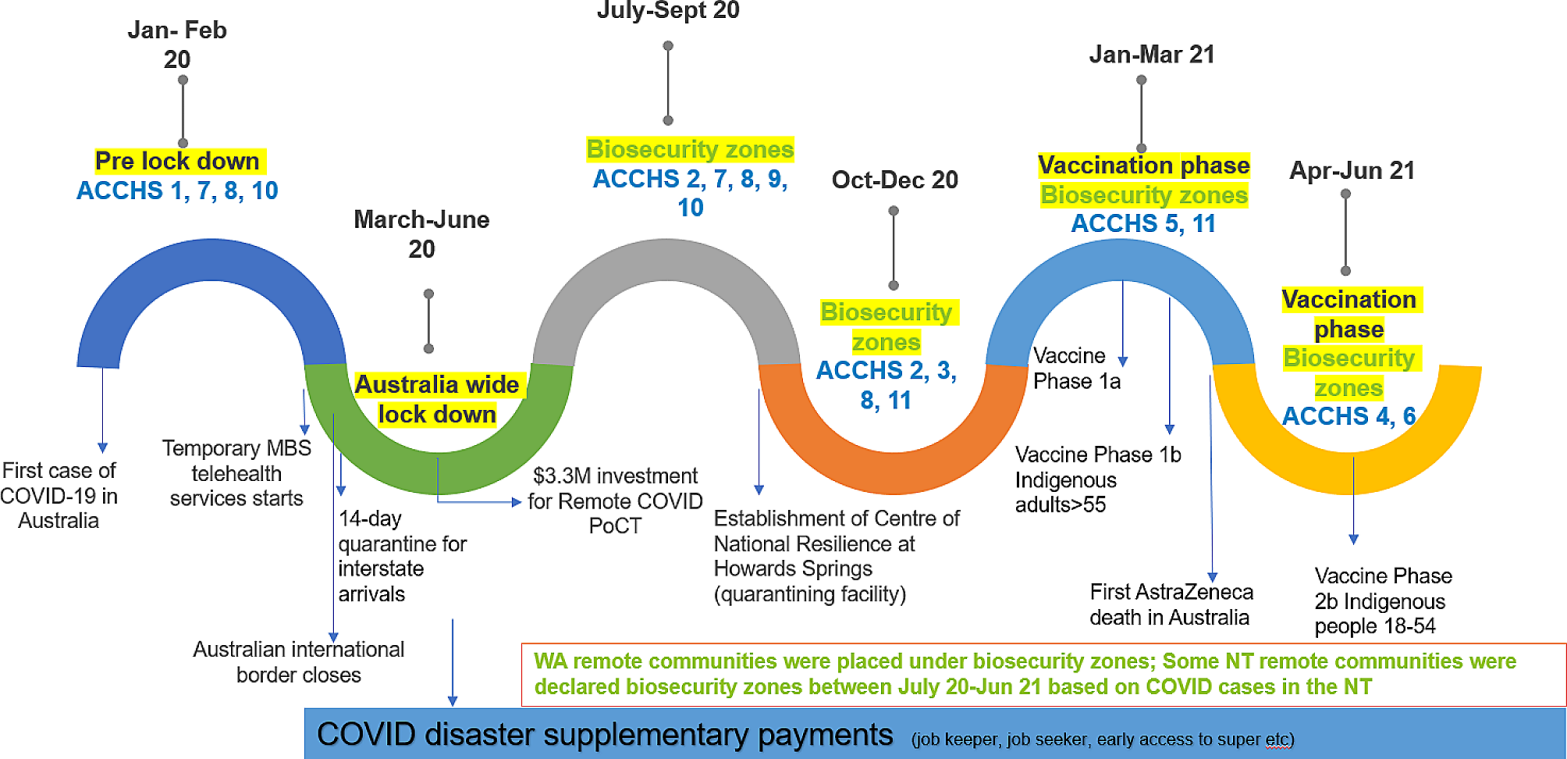
Dynamic policy landscape between February 2020 (start of data collection) and June 2021 (last month of data collection) relevant for primary health care service delivery in outer regional, remote and very remote Australia. Time periods at which data was collected from each ACCHS have also been marked. Sources: [16–18]

During the initial stage of the COVID-19 period from 2020 to 2021, regional and remote PHC clinics and communities were severely affected by travel restrictions (e.g. lock downs, declared biosecurity zones including the need to stay in quarantine before entering specific communities), which effectively prevented the spread of the virus. This was combined with the additional stress created by the lack of capacity for health staff to take annual leave due to both workforce shortages and lockdowns [11]. Despite clear benefits, the implementation of various strategies by the Australian Government such as funding for Point of Care Testing (rapid laboratory diagnostic results conducted at the site of patient care [12]), additional social security payments, and funding to promote telehealth (see Fig. 1) [7] was an unintended source of increased burden on health services staff [13]. A national survey of Australian PHC nurses with around 30% of participants from rural or remote locations found that many nurses perceived an overall reduction in the quality of usual care delivered during the COVID-19 period due to lack of time, supervision and decreased administrative capability [14].

Access issues and high workforce turnover and staff shortages existed pre-COVID-19 in remote Australia [8], the pandemic however further exposed the exacerbation of these workforce challenges, particularly due to the pre-existing reliance on agency nurses and fly-in-fly-out staff. It is thus important to document learnings from how the pandemic directly or indirectly impacted PHC delivery and service utilisation in regional and remote communities during this time to inform future planning of pandemic responses. In this paper, we synthesise PHC staff observations on PHC service utilisation and delivery in outer regional, remote and very remote communities during the COVID-19 preparation period.

## Data and methods

This paper sits within a broader study that explores the impact of short-term staffing on PHC delivery and clinic users [15]. COVID-19 occurred during the data collection of the broader study, providing opportunity to explore the effects of the pandemic on the PHC use and delivery. Semi-structured interviews were conducted with staff employed by 11 ACCHSs in the Northern Territory (NT) and Western Australia (WA). These ACCHSs deliver services in outer regional, remote and very remote communities, as defined by the Australian Bureau of Statistics Remoteness Areas classification [1]. The sizes of Aboriginal population serviced by the ACCHSs ranged from very remote communities with population under 100 to outer regional communities with a population of more than 10,000 people. The interview guide for the broader study is available as supplementary file 1. Data were collected between February 2020 and June 2021 and aligned with: (i) the pre-COVID planning period (February 2020 – mid March 2020), (ii) the Australia wide lockdown period (March-June 2020), (iii) the subsequent period where several communities across the NT and WA were declared as biosecurity zones due to the emergence of COVID-19 cases (July 2020 – June 2021) and (iv) the commencement of the vaccination roll out period (February 2021-June 2021) (see Fig. 1).

Research team members, including at least one Indigenous researcher visited each clinic and interviewed staff about the impact of short-term workforce on the service and the community. Interviews were recorded and the audio-recordings were transcribed verbatim. Interview transcripts were coded using NVivo v12 software (QSR International). One researcher read the interviews and coded the content inductively around common codes relating to PHC delivery and use during the COVID-19 period. Patterns in the codes and links between the codes were identified and organised into meaningful themes [19]. For each health service, three interviews were co-coded by two researchers (one Indigenous and one non-Indigenous researcher) and compared to validate the main themes. Any discrepancies were discussed with the wider research team until consensus was reached.

A steering committee that included representatives of the partnering health services and peak health bodies was established at the start of the project. Bimonthly written updates were sent to inform the committee about the progress of the project. The committee met every 6 months where members of the committee provided feedback on the findings that were presented by research team members. A detailed report of the findings was prepared for each health service. Feedback from each health service was collected which also informed the interpretation of the findings.

## Ethics

The study had ethics approval from the Human Research Ethics Committee of the Northern Territory Department of Health and Menzies School of Health Research (project number DR03171), Central Australian Human Research Ethics Committee (CA-19-3493) and Western Australian Aboriginal Health Ethics Committee (WAAHEC-938).

## Results

In total, 248 staff consented and participated in interviews, of whom 33% identified as Indigenous (also see Table 1). Diverse issues were discussed by staff across the themes of service utilisation (how remote clinics were used), PHC service delivery (how health services or clinics responded to community needs) and dissemination of information during the COVID-19 period (through what channels and with what impact).

**Table 1:** Number of participants against various professional categories

Administrative staff *	RANs	GPs	AHPs	Visiting specialists	Allied health staff	Other health workers *	Physical grade staff *
91	75	11	13	2	4	44	8

*Administrative staff included reception staff, finance officers, human resource staff, policy officers, managers, leadership staff and local health board members; Aboriginal community liaison officers and Aboriginal health workers were included in the other health worker category as unlike AHPs they are not registered with the Australian Health Practitioner Regulation Agency (AHPRA) and their roles have varied across the participating ACCHSs. Other health workers included Aboriginal community liaison officers, Aboriginal health workers, enrolled nurses, continuous quality improvement officers, health promotion officers, counsellors and physical grade staff included drivers, cleaners, gardeners, tradesmen and maintenance staff.

### Service utilisation

During the early phases of the pandemic, i.e. the lock down period and the biosecurity declaration periods, clinic staff observed changes in both how busy clinics were and the types of conditions that were presented.

#### Quieter clinics

Staff in most remote clinics reported that during the initial lockdown period the clinics were generally quieter than usual. Some staff suggested that there were also fewer acute respiratory presentations than what would otherwise be expected:

*“[There were] a lot of people who didn’t come to the clinic, like it was very quiet here” (ACCHS 1, AHP 99, Indigenous)*.

*“I would expect it [respiratory illness] would be a lot more than what we have been seeing because even when I’ve worked in small emergency departments, we see a lot more people with a cold than we have been here”* (*ACCHS 11, RAN 52, non-Indigenous*).

Staff indicated that decreased use of the service could be for a range of reasons –

First, some staff suggested that COVID-19 awareness programs and education about hygiene practices might have had an effect:

“… *washing hands, keeping their distance, all that kind of stuff, I don’t know whether this has really contributed but there is a noticeable difference” (ACCHS 11, RAN 56, non-Indigenous).*

Second, some staff indicated that patients might have been presenting earlier with mild symptoms and being treated before conditions became worse:

*“It’s hard for me to attribute to one specific thing but I’m thinking maybe people are just a bit more alert now and they come to clinic much sooner because of, they think it might be Corona[virus], is it this, and then they present earlier to clinic and they don’t just present late at night with, complications and difficult to treat issue” (ACCHS 11, RAN 52, non-Indigenous).*

Third, others felt people were not presenting to the clinic due to worries related to COVID testing or due to risk of transmission or fear that that they may be identified as the person identified as bringing COVID-19 to the community:

*“Whether that’s because people don’t want to come up … they know they’ll be swabbed…” (ACCHS 11, RAN 52, non-Indigenous).*

Fourth, border closures and travel restrictions into and out of remote communities were postulated to have reduced the usual high mobility of remote resident populations and thereby reduced the usual spread of respiratory infections:

*“I did notice it has been a bit quieter, but that’s about it. And there has been a lot to do with border closures and borders reopening. Like we’ll get a huge influx when people are coming back into town and having health checks and things like that, and then people go back out” (ACCHS 1, RAN 87, non-Indigenous).*

Finally, some staff speculated that COVID was associated with increased social cohesion which in turn may have led to decreased need for PHC:

*“COVID has been a Godsend for the community … everyone was sort of here and then, then they started doing things together. People started coming out at night and there’d be fires and there’d be singing and there’d be groups of people everywhere” (ACCHS 9, RAN 320, non-Indigenous*).

Staff raised concerns that inappropriately low utilisation and limited PHC services being available in some communities (some clinics had to close due to staff shortages) could result in more severe chronic conditions into the future increasing demand on medical retrieval.

*“There’s probably just groundswell of conditions and problems that are not being addressed. ….it’s just building up there, but we don’t know it, it’s going to hit us, people who would have been picked up as diabetics who are pre-diabetics won’t be identified. So, probably in another two or three months you might have … an acute presentation of somebody who’s sugar is way out of control” (ACCHS 4, Other health worker 13, non-Indigenous).*

#### Busier clinics

In contrast, in some communities staff reported that there were more clients using the health service because there were more people in the community as a result of the ‘Return to Country’ [20] policy (policy and health promotions that encouraged Aboriginal people to return to their usual place of residence) coupled with the biosecurity restrictions which meant once people returned they could not then leave their communities to travel back to regional centres. This was introduced to try and limit the transmission of the virus and keep remote communities safe [7]. Staff commented on the resultant issue of increased overcrowding in houses, which not only added to the “*tensions of people living together in a small area*” *(ACCHS 11, GP 53, non-Indigenous)*, but exacerbated the transmission of other non-COVID communicable diseases (e.g., skin infections), which had an impact on clinic utilisation. Staff felt that certain skin related diseases commonly observed among children weren’t presented in a timely way due to the fear of being reported to the government for child neglect:

*“They don’t present because they’ve got the fear that we’re going to report them to [to the government department that looks after child neglect] .you try living . with 15 people in your house. we had seen probably a 20% increase [in skin diseases] over the COVID [period] in [remote] populations” (ACCHS 11, RAN 51, non-Indigenous).*

Staff reported that some community members were anxious about the virus and visited the clinic for reassurance: “…*were coming in every single day, and they were anxious and worried about it [COVID]. They just wanted to talk about it” (ACCHS 9, Other health worker 315, non-Indigenous)*. Staff reported that some community members were diligent and keen to take COVID tests if they had symptoms, especially because they were concerned about spreading the disease to the community or their family members.

*“[A] lot of the community was worried. They’d come and say, “Can you swab me because I’ve got a sore throat”, … they were scared they were going to spread it to their family” (ACCHS 11, Administrative 57, non-Indigenous)*.

Mental health related presentations increased, according to clinic staff in some communities, due to people losing jobs, the inability to move around freely, shop for essential items ( e.g. winter clothes, new born clothes for their growing children) or access the nearest town: *“…the feeling that they couldn’t move, that they couldn’t go into town …release the valve, and I saw a lot more people with mental health presentations than I would normally” (ACCHS 11, GP 53, non-Indigenous).* Staff remarked that there was “*an increase in mental health cases from people who … never really had any issues, barely accessed the clinic, who were having breakdowns*” *(ACCHS 11, RAN 51, non-Indigenous).*

Alcohol and domestic violence related presentations were also perceived to have increased in some communities. Staff speculated that increased alcohol related presentations may have been due to the availability of illicit alcohol and the ability to purchase it through the additional income that was available through various federal government policies (for many recipients the income doubled compared to the pre-pandemic period [21]), as well as limited presence of health promotion staff in the communities. Concerns were voiced that there was a shift from managing chronic disease to reacting to acute presentations.

*“I think also because of the amount of money that’s been available with Centrelink payments and stuff, we’ve had issues around alcohol and violence and so that’s resulted in a lot of the care being more acute rather than chronic, looking at your chronic diseases and your program [alcohol and other drug program] stuff, and because program staff weren’t there, .a lot of the program work’s gone down as well” (ACCHS 4, Other health staff 116, Indigenous)*.

### PHC service delivery

Participants discussed that PHC service delivery had to adapt to the new circumstances brought about by the pandemic. Service delivery changed to account for people’s apprehension about COVID-19 (e.g. fears of contracting the virus or vaccine hesitancy), COVID-19 restrictions (e.g. lockdowns and declaration of biosecurity zones) and new PHC service delivery policies that were introduced (e.g. telehealth MBS items). Staff offered observations about how clinics and health services adapted in order to continue to effectively deliver PHC services in the communities.

#### Clinic resource availability during COVID-19

Staff talked about how COVID-19 preparedness brought about changes to regular clinic processes, and highlighted limitations of available clinic resources and infrastructure and a greater need for additional human resources for screening patients for COVID-19 related symptoms prior to appointments. Clinic staff interviewed in the early stages of data collection, i.e. soon after the Australia-wide lockdown raised concerns about insufficient availability of Personal Protective Equipment (PPE): *“I don’t think we had enough PPE …it was only lucky we didn’t have any positive cases, otherwise we would have been in trouble.” (ACCHS 1, AHP 99, Indigenous)*. Thereafter, most staff responses related to the resources required for patient screening for COVID-19 and how clinics worked to reduce transmission by isolating people presenting with COVID-19 symptoms from people presenting with other health issues. Participants described a range of measures put in place, including screening all patients for recent or current COVID-19 symptoms, regular cleaning of surfaces, installation of calling bells/desk call bells in clinics to alert staff about patient arrivals, chairs placed apart to ensure social distancing. Some clinics modified clinic infrastructure to separate patients with respiratory symptoms possibly related to COVID-19 from those without: “…*an infectious side and a non-infectious side (of the building) and that we, we had to keep those patients that side, so we had to reform that, all our fencing and gating, and the way the patients flowed into the centre” (ACCHS 2, RAN 222, non-Indigenous).* Staff commented that it was difficult to maintain social distancing given the lack of space in many small clinics, particularly during specialist visits. In some communities, men-only areas in the clinics were modified and utilised for patients who needed to be tested for COVID: “*Men’s section is not open as [the space was] needed for point of care testing” (ACCHS 2, GP 219, non-Indigenous)*. Most staff talked about having either a nurse or receptionist at the front desk who was assigned the duty of screening patients using a screening questionnaire that included questions related to COVID-19 symptoms: *“We had a nurse who was sitting at the front desk and she would be screening patients and stuff like that” (ACCHS 11, RAN 56, non-Indigenous).*

#### Flexible PHC delivery to accommodate community’s varying needs and policy changes

In some communities, staff became aware that many patients with chronic diseases were not visiting the clinics regularly, so staff proactively took steps to ensure medications were delivered to people’s homes. For patients who were fearful of swab testing, an option to spit in a jar was offered as an alternative to nasal and throat swab tests: “*When you could spit in a jar and get sputum, you have the same thing and no-one’s terrified” (ACCHS 3, Physical 24, Indigenous)*.

Telehealth, whereby clients were assessed by clinicians who were not physically present but in contact by phone or video link was introduced/enhanced in most clinics during the lockdown period. During this period, face-to-face General Practitioner (GP) consultations were less available but instead community members were able to consult with their regular GP using telephone or videoconferencing.

*“[To overcome] our lack of GP coverage [in community] over COVID, we were able to implement our telehealth service a little bit better.” (ACCHS 5, Administrative 16, non-Indigenous)*.

Many PHC allied health programs (e.g. podiatry, diabetes educators, cardiac educators*)* and specialist visits (e.g. renal physicians, ophthalmologists) ceased during the initial COVID lockdown period. With the clinics becoming quieter, remote clinic staff commenced outreach activities to ensure usual PHC services, such as child immunisation, were delivered: *“…like it was very quiet here, so then we just went outreach. So, one person would stay here, then another person would be on outreach, to go and see patients in the home” (ACCHS 1, AHP 99, Indigenous)*. Another commented: *“a lot of it was outreach. [Staff] would go out and get the kids …… for immunisation away from the clinic” (ACCHS 11, Administrative 57, non-Indigenous)*.

Outreach activities were challenging as staff were instructed to wear masks and gloves which, along with the news about COVID related deaths, were creating panic among community members. One staff member recalled:

*“I was scaring so many people out there, that I took them [protective gear] off, because they were terrified of that, and only gloves and mask too, that was all….And you know, coming through the news, there was all these thousands of people dying all over the world.” (ACCHS 9, RAN 322, non-Indigenous)*.

After the Australia-wide lockdown period, staff observed that other services started going back to normal and they could return to focus on their usual portfolios:

*“We have moved our focus back to getting adult checks done, and looking for underlying conditions and things like that. But previously it was a little bit of a what are you here for, let’s treat that and, and kind of minimising peoples’ time in the clinic. ….So you know podiatry, diabetes, cardiac educators, they’re only just starting to come back now that the borders [biosecurity zones lifted] have reopened” (ACCHS 11, RAN 54, non-Indigenous).*

### Dissemination of information and its impact

Remote health professionals also discussed the development of culturally appropriate educational resources and innovative ways by which information was disseminated to community members and the obstacles faced during various stages of COVID-19, including the vaccination period.

#### COVID related information resources

Some clinic staff were confident about the internal communications they were receiving about COVID-19, while others commented that policies were constantly changing, and the information they received was inadequate. Staff talked about the speed of ACCHSs’ responsiveness to the evolving pandemic, citing examples of employing staff in newly created COVID-19 specific positions, who: *“made sure [staff] had access to all the key [COVID-19] training information, activities, ensure timely communication with staff and community members” (ACCHS 3, Other health worker 26, non-Indigenous).*

In the initial COVID-19 preparatory phase (the period between Australia wide lockdown and before vaccinations became available), staff from ACCHSs and Indigenous health peak bodies were focussed on communicating social distancing rules, COVID-19 safe hygiene practices (how to cover your mouth when coughing or sneezing, proper wearing of masks, disposing of tissues etc.) and COVID-19 symptoms monitoring.

Staff spoke about how ACCHSs and the peak bodies for ACCHSs developed their own COVID-19 posters, booklets, video clips and screening sheets for use in communities [22]. These resources included a lot of graphics and were used by staff to quickly convey targeted messages to clinic users with maximum effect, including during consultations.

*“We had a booklet on COVID…. it’s something like “Communicating to Community Members with COVID.” So I actually printed it up and laminated it and put it into one of these things, so everybody that came in to my consulting room…could quickly go through the booklet” (ACCHS 9, Other health worker 315, non-Indigenous).*

Some health service staff, however, suggested that COVID-19 preparation and processes to communicate to community members were slow to take effect.

*“I think the response to set clinics up to be COVID-safe was a little bit harder, and actually have really good plans in place at the clinic, with signage and processes, I think that probably took a little bit longer” (ACCHS 4, Other health worker 116, non-Indigenous).*

In addition to developing and distributing COVID-specific educational materials, remote clinic staff also identified a need to respond to the increased number of mental health related presentations. Mental health educational resources such as posters were developed for community members and were placed in public places (e.g. on public phones), where the messages, including who to contact for help, were readily accessible.

#### Many ways of channelling information

According to the staff interviewed, patients received information through a range of channels. In addition to the aforementioned locally developed printed materials, remote clinic staff also utilised local interpreters to talk in local languages to community members about COVID-19 and precautionary measures to be taken. Messages were broadcasted on local Indigenous radio such as the CAAMA radio and through each clinic’s televisions. Clinic users were encouraged to listen to the radio for information:

*“Even though we had interpreters who talked to the community, it wasn’t enough, ‘cause I’ve said to people, “You might [want to] listen to CAAMA (local Indigenous radio)?” They reckon, “No,” but I instructed them. “You listen to CAAMA, ‘cause that’s where you’re going to hear what you need to know, and it’s going to be in language, so it’s easy for you to comprehend. (ACCHS 1, Other health worker 914, non-Indigenous)*

Information was also disseminated in informal settings:

*“In some areas where they couldn’t provide us with 1.5 metre distancing space inside, the clients were moved out and we did deliver education sessions under the tree outside” (ACCHS 2, Other health worker 27, non-Indigenous).*

Pop up display stands detailing COVID safety guidelines, hand washing stands at public spaces and other creative strategies were used to ensure information dissemination without attracting crowds. Door-to-door campaigns were found to be an effective way to communicate the risks to community members, especially to elderly people.

*“…door to door campaign really worked for us. The initial plan was actually to just do a public event in park, pop up stands. But then the [leadership team] said no it will attract crowds so we can’t do that, so after that we just went door to door to all the [name of community] and all the people really appreciated that” (ACCHS 2, RAN 217, non-Indigenous)*.

Public events were planned initially, but cancelled due to concerns of attracting crowds and inadvertently spreading COVID-19. Staff instead pivoted to a more personalised and targeted approach to the public health activities, leveraging on existing relationships where possible to have the greatest impact: “*we had to make sure that the [door to door campaign] group had a mix of Indigenous, non-Indigenous staff and only certain staff was sent to the [site name], who’ve had relationship with somebody and knew those[site name], you know, so it had a better impact” (ACCHS 2, Other health worker 217, non-Indigenous).*

Remote clinic staff ensured that they initiated ongoing conversations with community members about COVID-19 safe practices during ad-hoc visits to the clinic, thereby reinforcing earlier messaging:

*“So just educating people constantly every time you see them in the clinic or wherever” (ACCHS 9, Other health worker 315, non-Indigenous)*.

Another staff member noted the two-way nature of public health communications, with staff listening carefully to community concerns in order to be able to effectively address those concerns and health services providing information through respected community members using local languages:

*“We had those really frank conversations with community and had great engagement and great feedback from community and there was lots of discussion that then came from community members and board directors in language with the community, about not sharing smokes and not sharing drinks and those sorts of things” (ACCHS 5, RAN 511, non-Indigenous).*

One of the health services sought feedback from clinic users regarding their information dissemination strategy during the first stages of the COVID-19 period. Staff recognised that information sharing was becoming monotonous and frustrating for community members, given there weren’t any positive cases in communities. Staff kept revising the resources to ensure it wasn’t repetitive for community members:

*“So the community members said, it’s just getting too much, we’re hearing too much about it, you know….we’re just getting bored with this all the information” (ACCHS 2, Other health worker 27, non-Indigenous).*

All health services highlighted the importance of having culturally appropriate information dissemination materials. Staff worked within teams who were responsible to ensure any messaging was culturally appropriate.

“*You have got to be culturally appropriate in the way you deliver those messages…., our public health team did a lot of work around that, and that was working also with schools and places like that, so we’re all saying the same thing”* (*ACCHS 4, Administrative staff 11, non-Indigenous).*

Having local Indigenous staff leading the development of local educational resources was critical for cultural appropriateness:

“*Because we are [an]Aboriginal controlled organisation, so everything is culturally appropriate and specific for each community, the staff who were here that are recruited from the community usually take the lead in any resources that we make and then go through the process of cultural competency approval” (ACCHS 2, Other health staff 27, non-Indigenous).*

##### Misinformation

Staff highlighted that community members were being misinformed about COVID-19 by what they saw on social media platforms and that this adversely affected the effectiveness of information they disseminated through the health services. This was especially evident during the vaccine roll out stage. *“A lot of their education stuff comes from the internet or, Facebook” (ACCHS 7, Other health staff 315, non-Indigenous)*. Staff discussed specific examples of misinformation accessed by community members through various media. One example was that Indigenous people would not be affected by COVID-19; “*COVID…was a white fella problem”* (*ACCHS 4*, *Administrative staff* 62, *non-Indigenous*); Australia is not affected by COVID-19; *“if I stay in the community, I won’t get COVID”* (*ACCHS 4*, *Administrative staff* 62, *non-Indigenous*). There were also examples of vaccine misinformation, including a general lack of trust in the vaccines and that COVID-19 related deaths were not due to the virus, but vaccine related deaths. Many were confused and anxious about the relative risks of dying from COVID-19 versus dying from a blood clot caused by a vaccine, in the context of extensive media coverage given to a small number of highly publicised deaths following Astra Zeneca vaccination.

*“I guess all of the deaths that people are hearing about on TV, that’s from the [*AstraZeneca*] vaccine and not from the virus. They’re worried about blood clots I suppose.” (ACCHS 5, RAN 37, non-Indigenous)*.

As a result, staff felt it was important to educate community members well before the vaccinations were available in communities, so that misinformation and vaccine hesitancy could be addressed. Staff gave specific examples of how media hype had increased vaccine apprehension and anxiety in community. In one instance, a community member was vaccinated for COVID-19 and re-presented to the clinic feeling unwell and desperately asking for the vaccine to be taken out of her body as she was worried, she would die. In general, staff commented that *“a lot of people were scared to come in [for vaccinations]” (ACCHS 6, Administrative staff 21, non-Indigenous).* In the end, a major community engagement strategy that included “door to door” health education utilising local Aboriginal people and nurses was rolled out.

## Discussion

This study is an important synthesis of the perceptions of a large number of PHC staff regarding PHC service utilisation and delivery in outer regional, remote and very remote clinics during different phases of the COVID-19 pandemic. The perceived decline in PHC use observed across the different phases is consistent with PHC utilisation data published by the Australian Institute of Health and Welfare (AIHW) for the period January 2020 to December 2021 (see Figures S1a-b, [23]) and several other studies [24, 25]. While studies have associated the reasons for decline in PHC utilisation during the early COVID-19 planning phase to limited PHC services offered, patient fear of getting infected and higher public health related workloads of clinic staff, our study suggested additional possible contributors. These included - reduced availability of alcohol in some remote communities which may have reduced alcohol-related presentations; more stable populations within communities due to travel restrictions that may have reduced conflicts within communities; better income support through the Australian Government’s COVID-19 payment schemes reducing financial stressors and lessening the severity of poverty; and community members ability to spend more time in local cultural activities and less time travelling out of community which may have facilitated better social and emotional well-being as a result of greater connection to Country, culture and community [26]). The decreased PHC utilisation during the data collection period may not necessarily indicate better health outcomes for remote communities, as longer-term health implications could become evident later in the form of increased mortality and morbidity rates.

Increased income through the Australian Government’s COVID-19 supplementary payments predominantly improved people’s lives [21], there were perverse outcomes noted in a few communities, as in some instances community members were able to buy more alcohol from sly grog sellers, despite the enforcement of various alcohol restriction/management plans [27]. The Return to Country policy [20], initiated in the early phase of the pandemic, assisted many Indigenous people to return from larger towns to their remote communities. This, in conjunction with COVID-19 related travel restrictions which limited their subsequent mobility, exacerbated of overcrowding in remote communities and concomitant adverse impacts on spread of infectious diseases (e.g. increase in skin diseases) and development of mental health issues (e.g. anxiety and depression). Insufficient housing and inadequate housing infrastructure [28] has been recognised as key issues for Indigenous communities and is further highlighted in this study as it challenges the ability to adequately manage any infectious disease outbreaks in remote communities.

In terms of ensuring access to GP services, telehealth had an increased role in PHC delivery, as GP visits to communities ceased [13]. Despite the increased availability of telehealth, staff worried that limited utilisation of PHC services during the initial COVID-19 preparatory stages would result in more frequent or more acute clinic presentations at a later date, with concerns of increased need for retrievals and increased hospital admissions for potentially preventable primary health care conditions in the future. These concerns are consistent with the PHC utilisation trends reported by AIHW for outer regional, remote and very remote clinics, where the number of regular clients peaked after June 2021 [23] and with a recent report that two-thirds of remote NT Health clients with at least one chronic condition hadn’t had GP chronic disease management within a 12-month period [29]. This signifies the need to explore the acceptability of telehealth in remote community settings before further roll out.

Health services that deliver health care to remote communities are underfunded [30] and face workforce shortages [8]. Health service staff, who were already experiencing excessively heavy workloads, were required to adapt to fluctuations in the numbers and types of PHC presentations (e.g. increased screening for COVID-19), to the cessation of site visits from Allied Health and medical professionals, to the emergent imperative to conduct a range of public health activities such as developing contextually appropriate consumer resources, remodelling clinic infrastructure, educating and delivering vaccines, and to deliver services through outreach models when community members feared attending the clinic. The lack of adequate human and financial resourcing greatly impedes remote health service capacity to respond effectively and quickly when new threats such as the COVID-19 pandemic emerged. More investments are required to ensure remote clinics are adequately (human) resourced, and staffing models include some surplus capacity to support adequate responses to emerging health threats [31].

PHC delivery was affected by limited infrastructure to support best practice for screening and treating patients who presented with COVID-19 symptoms. Limited clinic space was an issue even before the pandemic. Some clinics utilised spaces that were previously dedicated for men’s health as COVID-19 screening spaces, which impacted on cultural acceptability for men seeking care. This can have longer-term adverse impacts on male health outcomes, and is highly undesirable as Indigenous men already have low health service utilisation rates [32]. There is a clear need for investing in fit-for-purpose and culturally suitable clinic infrastructure in remote communities.

Information dissemination related in initial phases to COVID-19 preparedness and in later phases to vaccinations was a significant focus of PHC delivery in remote clinics. Our study found that the planning and initial vaccination phases of the COVID-19 period impacted PHC utilisation in different ways in remote Australia. Indigenous leadership was considered by community member to be effective and receptive to their views when disseminating the information in the pre-vaccination stages [7]. The vaccination phase was reported by respondents to be challenging. Respondents perceived that social media was heavily influencing vaccine uptake/hesitancy; this was also found around the globe [33]. In particular, the news linked to AstraZeneca blood clots and subsequent misinformation was said to be a barrier to COVID-19 vaccination uptake amongst populations living in remote communities. This was also the case in other regions where AstraZeneca vaccine weren’t offered as Pfizer mRNA vaccines were readily available. During the vaccination phase, a door to door personal communication strategy proved to be successful. High vaccine coverage rates were achieved prior to opening of the NT borders at the end of 2021. By the time of the rapid spread of the Omicron variant in early 2022, the remote population was highly vaccinated [34]. This suggests that for the success of future vaccination campaigns, it is pertinent that all levels of government monitor varying public sentiments, including early identification of miscommunication through social media and respond quickly in ways that promote local understanding [33].

In summary, we found variability across communities in how they responded to COVID-19 and how various policies affected communities, and the impact on use of PHC services. Findings from this study, reveal the need to better align policies to the diversity of remote communities to mitigate future health crises; policy options based on local responses and knowledge are important considerations.

### Future research directions

In terms of future research directions, a quantitative analysis of the PHC utilisation trends pre COVID-19, during and post COVID-19 period stratified by remoteness, clinic location, age, gender, cause and acuity of presentation could reveal specific utilisation trends and insightful information into the effects of specific policies on remote communities.

### Strengths and limitations of the study

A key strength of the study is that it recorded perceptions of close to 250 staff in real time starting just before the lockdown till the start of the roll out of vaccines in remote communities. Interviews were conducted over a 17-month period and hence the focus of the responses varied depending on the COVID-19 phase at which the interview was conducted. This means staff who were interviewed in the initial stages weren’t able to provide their responses on issues emerging during later stages of the pandemic such as vaccine hesitancy. The research team included Indigenous researchers, but it is possible that cultural and language differences across the locations could have affected some of the staff interviews. The paper does not report the perceptions of community users, except when staff were also local community members.

## Conclusion

The differential effects of the pandemic on remote and very remote communities highlights the diverse needs of the communities. It is important to ensure ACCHSs are funded adequately to be able to adapt clinic infrastructure and service delivery depending on future pandemic circumstances, building community resilience and independence, while reducing morbidity and mortality. The importance of tailoring staff, resources, clinic infrastructure and culturally appropriate information to enable ACCHs to connect with their communities and continue their work cannot be overstated. For future pandemics, policy makers need to connect more closely with the remote community context and be better informed about the potential effects of new policies on remote health service delivery and heath service demand, to enable ACCHSs to maintain adequate levels of PHC.

### Electronic supplementary material

Below is the link to the electronic supplementary material.

Supplementary Material 1

Supplementary Material 2

## Data Availability

Ethics approvals were obtained from Human Research Ethics Committee of the Northern Territory Department of Health and Menzies School of Health Research (project number DR03171), Central Australian Human Research Ethics Committee (CA-19-3493) and the Western Australian Aboriginal Health Ethics Committee (WAAHEC-938). All methods were carried out in accordance with the guidelines set by the three ethics committees. Informed consent was obtained from all participants of this study. Consent was obtained only to publish aggregated data and not for individual level identified data from the participants. The qualitative data collected was thus de-identified and aggregated before analysis. De-identified quotes have been included in the paper.

## Abbreviations

PHC: Primary Health Care
ACCHSs: Aboriginal Community Controlled Health Services
MBS: Medicare Benefits Schedule
GPs: General Practitioners
NT: Northern Territory
WA: Western Australia
PHC: Primary Health Care
AHPs: Aboriginal Health Practitioners
RANs: Remote Area Nurses
NTG: Northern Territory Government
AIHW: Australian Institute of Health and Welfare
