# Supplementary material for: Primary health care utilisation and delivery in remote Australian clinics during the COVID-19 pandemic
Source: BMC Prim Care. 2024 Jul 5;25:240. doi: 10.1186/s12875-024-02485-3 (PMC11225297; doi:10.1186/s12875-024-02485-3)
Supplement: Supplementary file 1 — Supplementary Material 1 [file 12875_2024_2485_MOESM1_ESM.docx]

**Supplementary file 1**

Semi-structured interview protocol

1. For the purposes of the study, we want to know what proportion of the clinic staff we interview are of Aboriginal and Torres Strait Islander background. **Are you of Aboriginal and/or Torres Strait Islander background?** (Yes, Aboriginal; Yes, Torres Strait Islander; Yes, both Aboriginal and Torres Strait Islander; No; Do not want to answer)

2. **Can you tell me a bit about yourself and how you came to be working here?**

- Also explore participant’s rural/remote exposure/background prior to joining the clinic?
  - - - - Your training/preparation prior to starting here (including specific cultural safety training, qualifications/credentialing etc.)?
        - What settings were you working in before here?
        - What attracted you to work here (motivations)?
        - What were your expectations when you came here?
        - Were your expectations matched by the reality?
        - How long have you worked at the clinic/ health service?
        - How did you hear about this position?

3. **Can you tell me about your employment and role in this health clinic?**

- Are you permanent/contract/casual/agency?
- What is your role here?
- How long have you been working here?
- Is this your first time working at the clinic

4. **Can you tell me about this health clinic?**

- What do you find works well here? Why? Examples?
- What things are more difficult about working here? Why? Examples?

5. **Clinic staffing**

- How is this clinic staffed (currently)?
- How do you think this clinic should be staffed?
- Can you explain to me how this clinic uses short term staff?
- For planned staff relief (annual leave, cpd)?
- To fill in for long periods of time when unable to recruit to position?
- In different individuals each time vs return visits?
- For after-hours relief?
- For primary health care?
- What do you like about having short-term staff come in?
- What do you not like about having short-term staff come in?
- Are there any challenges associated with short-term staff?

6. **Staying in this clinic**

- How would you rate your job satisfaction from 1 to 10 (1 = very dissatisfied to 10 = very satisfied)?
- Do you think you’ll keep working here? If so, for how long?
- What keeps you working here?
- employment arrangements –contract/permanent position etc.?
- connections to people?
- connections to place?
- scope of practice?
- Are there any things that lead you to think about leaving?
- Is there anything the health service does for employees like you to keep you here?
- What additional things could be done to encourage you to stay longer?

7. **Recruiting and retaining staff to this health service**

- In your view, who are the right sort of staff to work here?
  - - Professional attributes?
    - Personal attributes?
    - Cultural or other attributes?
- What sort of strategies are being used here to recruit staff? When were they brought in? (permanent vs contract? Bonuses? CPD?) What is your sense of their effectiveness?
- What sort of strategies are being used here to keep staff? When were they brought in? (permanent vs contract? Bonuses? CPD?) What is your sense of their effectiveness?
- Do you have any suggestions about what else this health service could do to recruit the right sort of staff to work here?
- Do you have any suggestions about what could be done here to keep the right sort of staff?
- What pathways does the service have for its staff and patients to provide feedback about working or using the clinic?
- What has been your experience providing this feedback to the service?
- How does the health service use this information? Give examples.

8. **We are interested in any differences between community control of health services and government control.**

- What is your personal experience of working in communities with community and government control or clinics that have transitioned from government to community control?
- Do you think there are any differences between community control and government-run health services? Can you explain?

9. **Impact of COVID-19 on clinics and the ACCHS sector**

- The first cases of COVID-19 in Australia were confirmed at the end of January 2020. What were the challenges of delivering primary health care at your clinic resulting from COVID-19?
  - - Workforce challenges such as sourcing staff with remote skills and experience, loss of local staff; workload challenges, 14-day quarantine of interstate staff;
    - availability of resources including sourcing PPE, delays in point of care testing;
    - planning for COVID-19 (e.g. communication, screening, use of information resources) and vaccine uptake (e.g. misinformation)
    - changes in community member use of the clinic – such as reduction in the clinic use, changes in presentation types, less/more retrievals;
    - other changes within the community that may have impacted people’s health (e.g. food availability in store, increased reliance on bush tucker, some residents moving to smaller outstations/homelands, alcohol use);
    - unplanned consequences;
- Can you describe what responses your clinic and service developed to address these challenges?
  - - new policies to protect and support the existing workforce;
    - new ways of delivering PHC (e.g telemedicine);
    - has there been any change in telemedicine use? What have been the positives and negatives?
    - new positions;
- What lessons can be learnt by the responses of your service and the ACCHO sector to COVID-19?
- What will be the workforce challenges for the Aboriginal Community Controlled Health Service sector in the future related to COVID-19? Can you provide examples?
- How can these challenges be managed, monitored and addressed?
  - - by the clinic and the service;
    - the Aboriginal Community Controlled Health Service sector;
    - government;

10. Is there anything else that we haven’t discussed that you think is important?
