# Supplementary figures and images for: Primary health care utilisation and delivery in remote Australian clinics during the COVID-19 pandemic
Source: BMC Prim Care. 2024 Jul 5;25:240. doi: 10.1186/s12875-024-02485-3 (PMC11225297; doi:10.1186/s12875-024-02485-3)

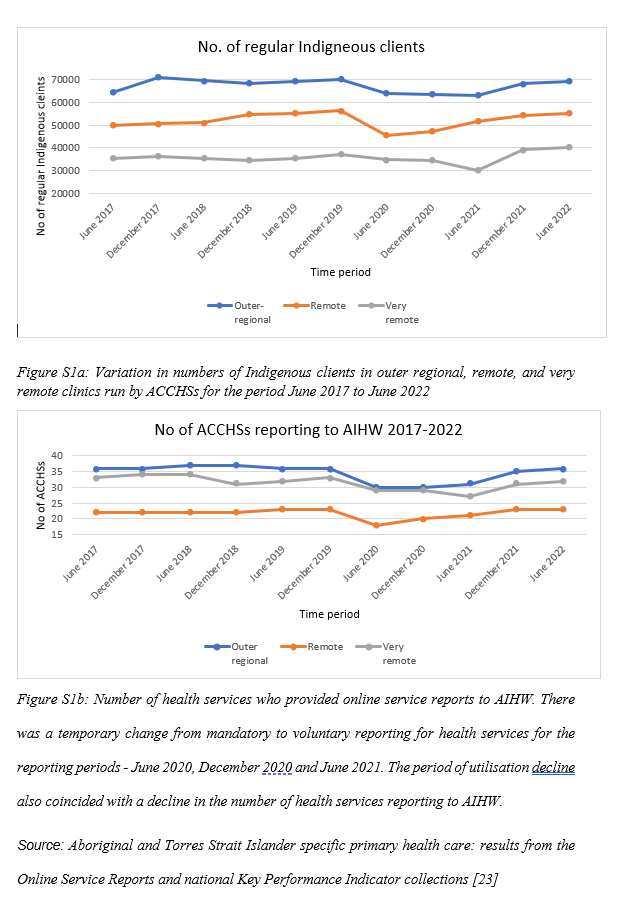

Supplement: Supplementary file 2 — Supplementary Material 2 [file 12875_2024_2485_MOESM2_ESM.png]
